# Lean body weight‐adjusted intravenous iodinated contrast dose for abdominal CT in dogs reduces interpatient enhancement variability while providing diagnostic quality organ enhancement

**DOI:** 10.1111/vru.13122

**Published:** 2022-06-10

**Authors:** Jennifer Kan, Marjorie Milne, Dayle Tyrrell, Caroline Mansfield

**Affiliations:** ^1^ Diagnostic Imaging Department of U‐Vet Werribee Animal Hospital Victoria Australia

**Keywords:** adipose tissue, dogs, iodine, iohexol, lean body weight, tomography, X‐ray computed

## Abstract

Contrast‐enhanced computed tomography (CECT) is increasingly used to screen for abdominal pathology in dogs, and the contrast dose used is commonly calculated as a linear function of total body weight (TBW). Body fat is not metabolically active and contributes little to dispersing or diluting contrast medium (CM) in the blood. This prospective, analytic, cross‐section design pilot study aimed to establish the feasibility of intravenous CM dosed according to lean body weight (LBW) for abdominal CECT in dogs compared to TBW. We hypothesized that when dosing intravenous CM according to LBW, studies will remain at diagnostic quality, there will be a reduced interindividual contrast enhancement (CE) variability, and there will be less change to heart rate and blood pressure in dogs compared to when administering CM calculated on TBW. Twelve dogs had two CECT studies with contrast doses according to TBW and LBW at least 8 weeks apart. Interindividual organ and vessel CE variability, diagnostic quality of the studies, and changes in physiological status were compared between protocols. The LBW‐based protocol provided less variability in the CE of most organs and vessels (except the aorta). When dosed according to LBW, liver enhancement was positively associated with grams of iodine per kg TBW during the portal venous phase (*P* = 0.046). There was no significant difference in physiological parameters after CM administration between dosing protocols. Our conclusion is that a CM dose based on LBW for abdominal CECT lowers interindividual CE variability and is effective at maintaining studies of diagnostic quality.

**Abbreviations**
CE: contrast enhancement
CECT: contrast‐enhanced computed tomography
CIN: contrast‐induced nephropathy
CM: contrast media
DEXA: dual‐energy X‐ray absorptiometry
LBW: lean body weight
ROI: region of interest
TBW: total body weight

## 1 INTRODUCTION

Contrast‐enhanced computed tomography (CECT) is increasingly being used to investigate abdominal pathology in dogs, and the main technical parameter influencing solid visceral enhancement is the amount of iodine administered, with solid visceral enhancement increasing proportionally with iodine concentration.^1^ The contrast medium (CM) dose is commonly adjusted according to the patient's total body weight (TBW), as it is simple to calculate and can be readily implemented to achieve adequate organ opacification.^2^ However, CM dosage according to TBW fails to consider differences in body conformation, and because fat is poorly perfused and less metabolically active, TBW calculated regimens may lead to excessive contrast volume in overweight and obese patients.^3^

Contrast enhancement (CE) during CT examination is affected by multiple factors related to the patient, CM, and CT scanning technique.^2^ The important technique‐related factors include CM volume, concentration, rate of injection, and type of injection.^4^ In humans, key patient‐related factors affecting CE are patient body size and cardiac output (cardiovascular circulation time), and among body size parameters, lean body weight (LBW) exhibits the strongest association with aortic and hepatic enhancement.^5^ Excluding disease states that alter circulation, such as congestive heart failure, the most important patient‐related factor is body weight.^4^ The minimally sufficient iodine dose to achieve adequate parenchymal and vessel CE should be given to reduce any potential toxicity or adverse alteration to physiological functions.^2^ In dogs, the empirically recommended dose for abdominal CT is 600 to 880 milligrams of iodine per kilogram (mgI/kg), which is higher than that for human adults (600 mgI/kg).^3^, ^6^ Goic and coauthors found that contrast‐induced kidney injury occurred in dogs administered a median contrast dose of 897.4 mgI/kg, just above the recommended dose range.^7^ Acute severe reactions to intravenous contrast are rare, although moderate (>20% from baseline) bradycardia, tachycardia, hypotension, or hypertension when Iohexol was administered in 18.3% of anesthetized dogs have been reported.^8^ Use of less iodinated contrast is particularly crucial in patients with preexisting renal dysfunction, dehydration, hypertension, or on nephrotoxic chemotherapeutic regimens; those patients have a higher risk of developing contrast‐induced nephropathy (CIN) as CM is mainly excreted by the kidneys unmetabolized.^2^, ^9^ Lean body weight tailored contrast dosage has been studied in humans, as an obese patient has a high proportion of body fat and relatively small blood volume and extracellular compartment; the higher proportion of fat contributes little to dispersing or diluting CM in the blood.^2^, ^10^ It has been reported in people that LBW‐adjusted contrast dose reduces patient‐to‐patient enhancement variability while maintaining satisfactory hepatic and vascular enhancement.^3^, ^11^, ^12^, ^13^ In a recent study, a CECT protocol using 600 mgI/kg fat‐free mass provided diagnostic liver enhancement and improved interpatient enhancement uniformity while reducing iodine dose.^14^ Kondo and coauthors recommended a dose of 750 mgI/kg LBW to produce adequate hepatic enhancement, and Zanardo and coauthors suggested that diagnostic abdominal CT may be obtained using 630 mgI/kg LBW, with margins for dose reduction.^12^, ^15^ Such recommendations for adjusting CM dose according to LBW in dogs are currently lacking. A previous study found a positive association between abdominal fat percentage and CE of the aorta, liver, and portal vein during the portal venous phase.^16^ The positive association between aortic, hepatic, and portal enhancement was suspected to be due to a greater extracellular concentration of iodine in dogs with a higher abdominal fat percentage.^16^ Findings from this study introduced evidence that abdominal fat percentage is significantly associated with organ attenuation values during abdominal CECT studies in dogs, and calculations for contrast dose may need to consider body fat as well as TBW.

The aim of the current study was to determine whether an LBW‐adjusted contrast dose in CECT would have reduced interindividual enhancement variability, be of diagnostic quality, and produce fewer physiological changes in heart rate and blood pressure. We hypothesized that, when dosing CM according to LBW, there would be reduced interpatient enhancement variability, satisfactory organ/vessel enhancement, and image quality, as well as lower changes to heart rate and blood pressure following CM administration.

## 2 MATERIALS AND METHODS

### 2.1 Study design and subject recruitment

This pilot study was a prospective, analytic, cross‐sectional design involving a convenience sample of 12 dogs. All dogs were admitted to the U‐Vet Werribee Animal Hospital during the period of 11/7/2019 to 26/4/2021. The clinical indication of CECT in these dogs with suspected abdominal pathology was to improve delineation of major blood vessels, normal organ tissue, and pathological lesions, aiding in the diagnoses of abdominal and extra‐abdominal neoplasms and nonneoplastic diseases (e.g., pancreatitis). Approval for the use of animals was granted by The University of Melbourne Office for Research Ethics and Integrity (ID No: 1914822.1). Owner consent was provided prior to an animal's inclusion in the study, and the use of the data was approved by the hospital director. All dogs would have two abdominal multiphasic (arterial and portal venous phase) CECT studies as part of normal clinical investigations at U‐Vet Werribee Animal Hospital, a minimum of 8 weeks apart. The initial CECT study was acquired with intravenous CM dosed according to TBW. Dogs presenting for a second CECT scan at least 8 weeks later and recruited to the study had iodinated CM dosed according to LBW. The TBW and LBW dosed CT studies were performed at least 8 weeks apart, as a study in normal beagle dogs showed that there was no clinically significant effect of repeated CM administration after 6 to 8 weeks.^17^ The shortest time frame between studies was 8 weeks, and the longest was 58 weeks (median = 17.5).U‐Vet Werribee Animal Hospital

Dogs were included if they had normal renal parameters (serum urea < 8.7 mmol/L and creatinine < 140 μmmol/L) or normal urine specific gravity (>1.030), as renal insufficiency would affect contrast excretion and organ/vessel enhancement.^2^ Dogs were also included if their TBW did not vary by more than 20% between both CT studies and must have had their heart rate and blood pressure recorded before and after the administration of intravenous contrast. Dogs were included if the initial CECT used a fixed injection duration protocol as described by Thierry and co‐workers; iodinated contrast dosage of 700 mg I/kg TBW, contrast administration by a cephalic vein intravenous catheter, and a CT study performed with a fixed injection duration of 20 s (range of injection flow rate: 0.8‐6 ml/s), with the arterial and portal venous acquisition triggered 10 s and 35 s after aortic arrival, respectively.^18^ This protocol was chosen as Tsuge and coauthors showed that CM administered by a fixed injection duration protocol of 35 s resulted in improved solid visceral enhancement in the portal venous phase in people.^19^ Final decisions for subject inclusion were made based on a consensus of a veterinary radiology specialist (M.M., Australian and New Zealand College of Veterinary Radiology) and a third‐year radiology resident (J.K.).

### 2.2 CT study acquisition and LBW determination

All CT scans were performed with a 16‐slice helical machine (Somatom Emotion 16, Siemens, Erlangen, Germany; collimation thickness: 1.2 mm; interval index: 1.5 mm; pitch: 1.3; tube rotation time: 0.6 s; mA: 200 kVp: 100) with the dogs in sternal recumbency. Transverse image data, with the image field of view extending from the cranial diaphragm to the caudal pelvis (matrix: 512 mm x 512 mm), were reconstructed using soft tissue algorithms (width: 350 HU, level: 40 HU) and reformatted into dorsal and sagittal planes. Iohexol (Omnipaque 350 mg I/ml, GE Healthcare, Marlborough, Massachusetts) was administered by a power injector (Salient Injector Head Single, DC009S, Imaxeon Pty Ltd, Pymble, New South Wales). A bolus‐tracking protocol was used, with the arterial acquisition in a cranial to caudal direction. The arterial phase is triggered 10 s after attenuation within a region of interest (ROI), as large as the aortic lumen, placed in the aorta just cranial to the diaphragm reached 100 HU.

Lean body weight was calculated based on estimated abdominal fat mass from precontrast abdominal CT data using proprietary CT software (Somaris/5 Syngo CT 2014A, Siemens AG, München, Germany) and following the method described by Turner and collaborators^20^ ^.^ Abdominal fat volume was calculated as all tissue between −25 and −250 Hounsfield units (HU) extending between the cranial margin of the 10th thoracic vertebra to the cranial margin of the first sacral vertebra, and total dual‐energy X‐ray absorptiometry (DEXA) fat mass was estimated with the regression equation below.^20^

y=1.70x+1.35

*y*: total fat mass in kg estimated by abdominal CT volume.

*x*: abdominal fat volume in liters calculated by abdominal CT using the fat attenuation threshold range.

LBW was derived by subtracting the total DEXA fat mass from TBW, and all assessments and measurements were checked by J.K. Abdominal volume CT to calculate LBW as the method is shown to reliably estimate total body composition in dogs between 5.1‐60 kg TBW as determined by DEXA.^20^ The 12 dogs were administered 700 mgI/kg LBW through a cephalic vein catheter, with the same injection and acquisition protocol as the TBW‐dosed CT scan.

### 2.3 Image analysis

Images of the TBW and LBW dosed CT studies were reviewed by a veterinary radiology resident (J.K.) using dedicated DICOM viewer software (SYNAPSE v 4.4.360, FUJIFILM Medical Systems, Morrisville, North Carolina). Attenuation values were measured on a single transverse slice at the level of the coeliac artery, as described by Kan and coauthor, by placing a circular ROI, approximately 25 mm in circumference and 50 mm^2^ in area, in the visualized portion of the aorta, liver, spleen, cortex of the right kidney, and portal vein.^16^ Caudal vena cava attenuation values were not performed, as contrast streaming artifacts were often observed during the arterial phase. This was performed on the unenhanced CT images and contrast‐enhanced CT images during the arterial and portal venous phase, avoiding blood vessels in the liver and spleen, bile ducts in the liver, focal lesions in the liver, spleen, and kidneys, calcifications in the liver and kidneys and artifacts. Assessment criteria for structurally normal organs included unremarkable margination, homogeneity on pre‐ and postcontrast CT scans, and morphology of the vasculature. The degree of CE was calculated as the difference in the attenuation values between the unenhanced and contrast‐enhanced images.

Two ANZCVS (Radiology)‐certified veterinary radiology specialists (M.M., D.T.) who were blinded to the intravenous dosing method reviewed images using DICOM viewer software to subjectively assess organ/vessel CE and graded the CE as excellent, good, fair, poor or no CE. The radiologists were permitted to adjust window widths and levels at will and scored the degree of organ/vessel enhancement based on an ordinal scale adopted from Yamashita and co‐authors.^4^ Both observers provided images of the 12 dogs, six of which had intravenous contrast doses according to TBW and six adjusted for LBW. The radiologists were not given both studies (TBW and LBW doses) for each dog to grade to prevent bias if the abdominal pathology was recognized in the studies of the same dog. For each dog, the method of the intravenous contrast dosing study chosen for review was performed with a random number generator.

### 2.4 Statistical analysis

Statistical analyses were performed using commercially available statistics software (IBM SPSS Statistics version 27, IBM, Armonk, New York). The Shapiro–Wilk test was used to assess normality of distributions of the organ and vessel attenuation. Frequency and descriptive statistics were calculated for variables such as age, sex, TBW, LBW, abdominal fat percentage, iodine load, iodine per kg TBW, and organ/vessel enhancement. Parametric data were compared using paired sample t tests, and nonparametric data were compared with Wilcoxon signed‐rank tests. The Wilcoxon signed‐rank test was performed to compare the change in heart rate and systolic blood pressure, and a paired sample *t*test was performed to compare the change in mean arterial pressure after contrast administration in both dosing protocols.

Linear regression models were generated using organ/vessel enhancement as the outcome and iodine per kg TBW as the explanatory variable. Subjective scoring of organ CE by two radiologists was compared between the LBW and TBW groups. To assess interobserver measurement variability in the visual analysis, a Cohen's K test was implemented. Values ≤0 indicated no agreement and 0.01−0.20 indicated none to slight agreement, 0.21−0.40 indicated fair agreement, 0.41– 0.60 indicated moderate agreement, 0.61−0.80 indicated substantial agreement, and 0.81−1.00 indicated almost perfect agreement.^21^ A *P*‐value less than 0.05 was used to determine significance.

## 3 RESULTS

### 3.1 Demographic data

There were five female neutered and seven male neutered dogs. The study group consisted of a range of breeds, including two Boxers, two Staffordshire Bull Terriers, and eight other breeds. They had a median age of 7.5 years (range 1–13 years; interquartile range 5.0–9.5 years). Descriptive statistics of the 12 dogs are summarized in Table 1 and Supporting Information S1. There was no significant difference in the distributions of TBW, LBW, and abdominal fat percentage for dogs presenting for their first and second CT scans. Calculation of CM dose according to LBW did, however, produce a significant reduction in iodine contrast dose (absolute dose and dose per kg TBW).

**TABLE 1:** Descriptive data of dogs (n = 12) when dosed according to TBW and LBW. Significance based on a paired sample t test or Wilcoxon signed rank test if data were normally or not normally distributed, respectively

	TBW dosed scan	LBW dosed scan	Significance
**Iodine dose (g)**			
Mean	21.2	16.1	*P* < 0.001
Range	9.1–42.0	5.3–25.2	
(Standard Deviation)	(9.6)	(6.0)	
**Iodine dose (g/kg TBW)**			
Median	0.70	0.47	*P* = 0.002
Range	0.68–0.70	0.44–0.62	
(Interquartile Range)	(0.69–0.70)	(0.45–0.49)	

The following clinical diagnoses for the dogs were recorded: abdominal neoplasia (n = 4), focally affecting the liver, adrenal glands, gastrointestinal tract or urethra, and extra‐abdominal neoplasms (n = 6) involving the cutaneous tissues, pericardium or testicles. Two dogs had nonneoplastic diseases, including pancreatitis and suspected thoracic foreign bodies. None of the dogs had a history of renal disease or congestive heart failure.

### 3.2 Organ enhancement

**FIGURE 1:**
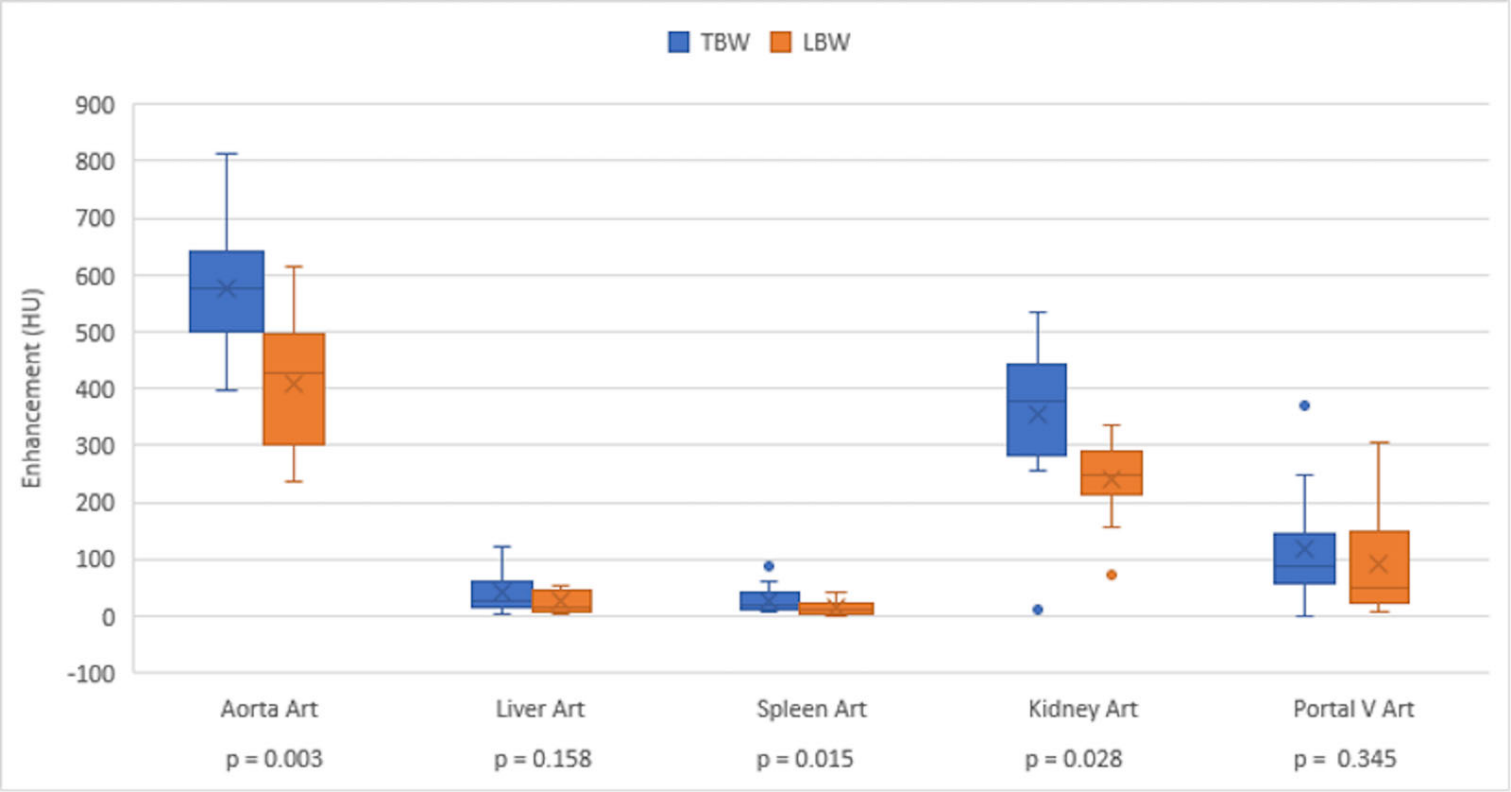
Organ and vessel enhancement during the arterial phase during the TBW and LBW dosed scans. HU, Hounsfield units [Colour figure can be viewed at wileyonlinelibrary.com]

**FIGURE 2:**
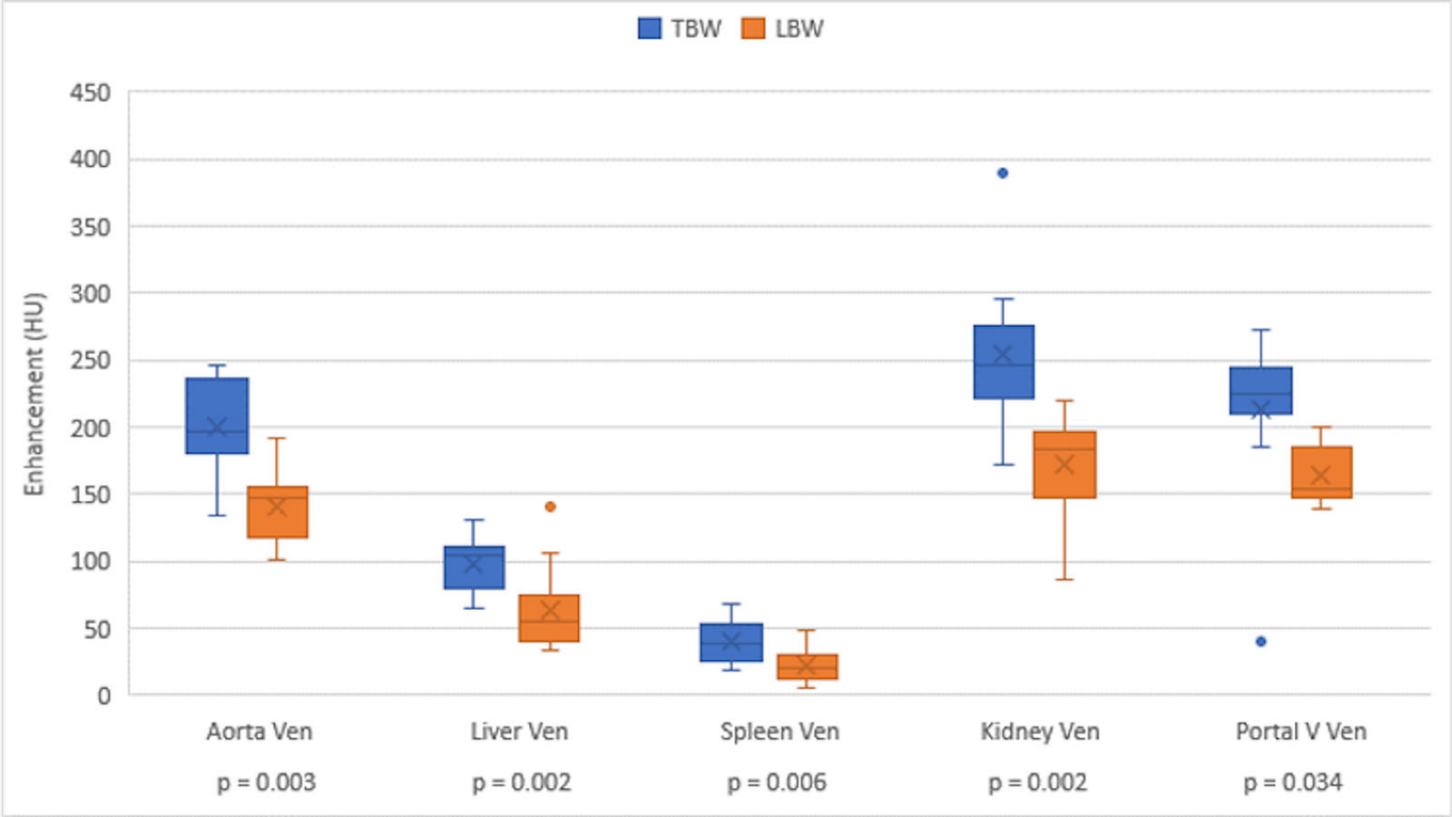
Organ and vessel enhancement during the portal venous phase during the TBW and LBW dosed scans. HU, Hounsfield units [Colour figure can be viewed at wileyonlinelibrary.com]

Box and whisker plots displaying data for CE of the aorta, liver, spleen, right kidney, and portal vein, comparing TBW to LBW dosing are shown in Figures 1 and 2. Figure 1 displays enhancement during the arterial phase, and Figure 2 displays enhancement during the venous phase. Supporting descriptive statistics are found in Supplementary 2.

The LBW dosing resulted in a statistically significant lower CE of organs and vessels, except for the liver and portal vein, during the arterial phase (*P* = 0.158 and 0.345, respectively). Despite this, a blinded review of LBW and TBW studies by radiologists rated image quality as good to excellent for all organs and vessels except the spleen, with unsatisfactory CE of the spleen identified in 4/12 LBW studies and 1/12 TBW studies (Table 2). Contrast enhancement in LBW studies was more frequently rated as good, while opacification in TBW studies was more frequently rated as excellent. The overall interobserver agreement for rating organ CE based on visual analysis by two radiologists was fair at 0.34. LBW dose‐adjusted studies had less variation in CE compared to the TBW group, except in the aorta during arterial and portal venous phases.

**TABLE 2:** Subjective evaluation of the quality of organ/vessel enhancement with intravenous contrast dosed according to TBW (n = 6) and LBW (n = 6) by two independent blinded radiologists

	No enhancement	Poor	Fair	Good	Excellent
Liver TBW	0	0	0	1	11
Liver LBW	0	0	0	4	8
Spleen TBW	0	0	1	2	9
Spleen LBW	0	0	4	2	6
Kidneys TBW	0	0	0	0	12
Kidneys LBW	0	0	0	1	11
Vasculature TBW	0	0	0	0	12
Vasculature LBW	0	0	0	1	11

**Excellent**: contrast enhancement provided optimal information to make a radiologic diagnosis. **Good**: contrast enhancement provided adequate information to make a radiologic diagnosis. **Fair**: contrast enhancement provided acceptable information to make a radiologic diagnosis, but image quality was unsatisfactory. **Poor**: contrast enhancement did not provide adequate information to make a radiologic diagnosis.

Findings based on a linear regression model (Table 3) showed a positive correlation between iodine per kg TBW for liver enhancement during the portal venous phase (*P* = 0.046). Hepatic enhancement tended to be positively linearly associated with iodine dose but did not reach statistical significance (*P* = 0.161). An association was not observed for splenic, renal, and portal venous enhancement.

**TABLE 3:** Linear regression analysis of relationships between organ/vessel contrast enhancement and iodine per kg TBW when contrast was dosed according to LBW

	Intercept (95% Confidence Interval)	Gradient (95% Confidence Interval)	Coefficient of Determination (*R* ^2^)	*P*‐value
**Arterial phase**				
Aorta	830.5	−873.9	0.085	0.186
	(164.7 – 1498.4)	(−2243.8 – 496.0)		
Liver	−54.2	163.1	0.186	0.161
	(−170.9 – 62.6)	(−77.0 – 403.3)		
Spleen	−5.3	41.9	0.032	0.579
	(41.9 – 73.0)	(−120.9 – 204.6)		
Kidney	331.8	−190.4	0.020	0.661
	(−124.8 – 788.5)	(−1129.9 – 749.0)		
Portal Vein	−102.4	399.7	0.050	0.485
	(−700.1 – 495.2)	(−829.9 – 1629.3)		
**Portal Venous Phase**				
Aorta	156.9	−33.0	0.005	0.834
	(−9.3 – 323.0)	(−374.8 – 308.9)		
Liver	−102.4	344.0	0.342	0.046*
	(−265.8 – 61.0)	(7.8 – 680.2)		
Spleen	11.4	21.6	0.212	0.771
	(−67.0 – 89.7)	(−139.5 – 182.8)		
Kidney	236.6	−133.2	0.038	0.542
	(8.2 – 464.9)	(−603.1 – 336.6)		
Portal Vein	193.6	−63.2	0.027	0.608
	(64.5 – 322.7)	(−328.8 – 202.5)		

**FIGURE 3:**
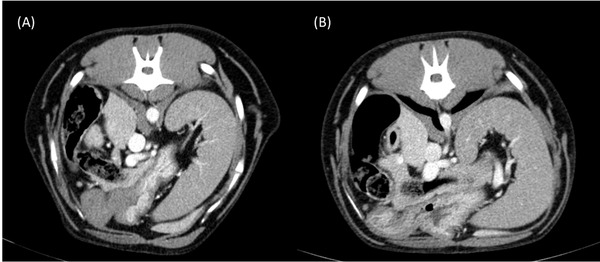
Transverse abdominal CT images obtained at the level of the celiac artery during the portal venous phase in the study dosed according to A, TBW and B, LBW. This patient was scored as having excellent contrast enhancement in all the assessed organs and vessels by both radiologists in the studies dosed according to TBW and LBW. Collimation thickness: 1.2 mm; interval index: 1.5 mm; pitch: 1.3; tube rotation time: 0.6 s; mA: 55 kVp: 110; soft tissue window width: 350 HU, level: 40 HU

**FIGURE 4:**
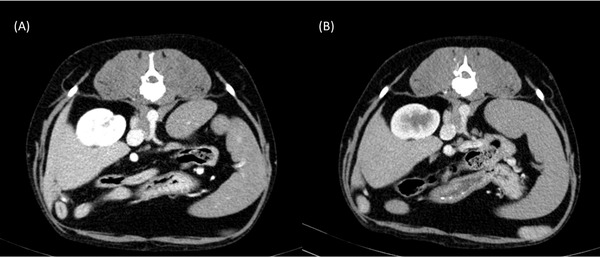
Transverse abdominal CT images at the level of the coeliac artery during the portal venous phase in the study dosed according to A, TBW and B, LBW. This patient had a hepatic enhancement of < 50 HU and was scored as having good hepatic enhancement by both radiologists when dosed according to LBW. Collimation thickness: 1.2 mm; interval index: 1.5 mm; pitch: 1.3; tube rotation time: 0.6 s; mA: 55 kVp: 110; soft tissue window width: 350 HU, level: 40 HU

In all dogs, the aortic CE exceeded 200 HU when dosed according to either TBW or LBW. Five of the 12 dogs in this study had hepatic enhancement < 50 HU when contrast was dosed according to LBW; however, they were still considered to have good to excellent CE by both radiologists on subjective visual assessment (Figures 3 and 4).

### 3.3 Physiologic parameters

**FIGURE 5:**
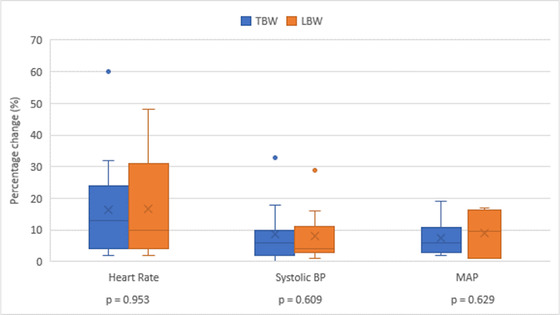
Percentage change in heart rate, systolic blood pressure, and mean arterial pressure before and after contrast administration when dogs are dosed according to TBW and LBW [Colour figure can be viewed at wileyonlinelibrary.com]

No significant difference was found when comparing heart rate, systolic blood pressure, and mean arterial pressure change after contrast administration between the two dosing methods (Figure 5).

## 4 DISCUSSION

This study found that LBW dosing of CM for abdominal CECT in dogs resulted in a reduced interindividual enhancement variability in most of the organs and vessels in the arterial and portal venous phases while maintaining adequate aortic enhancement (> 200 HU). These findings are consistent with a human study by Kondoand collaborators. and Yanaga and collaborators.^3^, ^13^ The resulting CE in all the dogs was sufficient to make a radiographic diagnosis based on visual assessment by radiologists. Contrary to our hypothesis, LBW dosing of CM did not produce a significant difference in the change in heart rate and blood pressure compared with TBW dosing of CM. Based on the authors’ review of the literature, no previous publications have described using LBW adjusted intravenous contrast dose for abdominal CT in dogs. The present study provides supportive evidence for dose reduction of CM in obese dogs while maintaining diagnostic image quality.

In people, patient‐to‐patient liver enhancement uniformity is essential for lesion assessment, accuracy, and reporting confidence in diagnostic and follow‐up CT examinations.^14^ Multiple human studies have explored the use of LBW as an alternative metric to TBW in CM dosing.^11^, ^14^, ^22^, ^23^, ^24^ These studies show contradicting results, with some reporting no difference in enhancement or interpatient variability between TBW and LBW dosing protocols.^23^, ^25^ Two studies showed that the use of LBW (rather than TBW) resulted in a more precise estimation of iodine dose to achieve consistent hepatic enhancement with reduced patient‐to‐patient variability.^12^, ^22^ Zanca and coauthors also found that a fat‐free mass‐based protocol yielded similar findings and significantly reduced iodine load.^14^ Based on reports in the human literature and findings from the present study, TBW may not be the optimal body size index for determining CM dose, particularly in obese patients with abundant body fat and thus relatively smaller vascular and interstitial spaces for their body weight.^3^

In the present study, when dosed according to TBW, all the dogs had hepatic enhancement > 60 HU; this would equate to overenhancement in humans.^14^ When contrast was dosed according to LBW, 5 of the 12 dogs in this study had hepatic enhancement < 50 HU, which is considered insufficient opacification in humans.^3^, ^4^, ^24^ However, the enhancement was above 30 HU in all the dogs, which is the cut‐off for inadequate enhancement in humans, when the conspicuity of lesions will be diminished.^26^ In the study by Zanardo and co‐workers, approximately half of the patients had a liver CE below 40 HU; however, they were considered diagnostic by radiologists.^25^ This was thought to be due to technological improvements in both CT hardware and software that decreased not only the ionizing radiation dose but also the dose of CM needed for a diagnostic examination, and the authors suggested that the threshold of 50 HU for sufficient liver CE should be reconsidered.^25^

In people, it was reported that approximately 0.5 g iodine per kg of TBW is required to achieve an average 50 HU increase in CE in the liver.^27^ In the present study, this dose was only achieved in three of the 12 dogs when the dose was adjusted to LBW. This could explain why visual assessment of organ enhancement recorded fewer “excellent” scores in dogs dosed according to LBW. Interestingly, for both contrast dosing methods, splenic enhancement was generally lower, being scored as “fair” once when dosed according to TBW and four times when dosed according to LBW. The reason for this is not known; however, quantitative splenic enhancement with the fixed injection duration protocol was overall lower compared with a fixed portal phase acquisition timing in the study by Kan and coauthor.^16^ This could be due to later performed portal studies when a fixed injection duration protocol is used.^18^ Or reflect anatomic peculiarities for the spleen such as the splenic pulp.

In people, liver CE is considered the reference for solid organ parenchymal CE, and it is strongly influenced by CM biodistribution into the intra‐ and extravascular space but appears to be a nonlinear multiparametric function of several variables that may not be modeled by simply using TBW.^25^, ^27^ LBW is a major predictor of functional capacity in the field of pharmacology, and Awai and collaborators suggest it as a determinant for the administration of iodine‐containing CM.^5^ In the present study, liver enhancement during the portal venous phase was positively associated with milligrams of iodine per kg TBW (*P* = 0.048) when dosed according to LBW. This supports the use of LBW to determine CM dose.

As such, LBW dosing of CM for CECT of the abdomen should be considered when studying dogs with extreme body composition, as dosing CM according to TBW could lead to overdosing in obese dogs or under dosing in thin/emaciated dogs. Reducing the dose of iodine in obese dogs may decrease the risk of CIN, as the incidence of this nephropathy is dose‐related.^2^, ^28^ Reduced CM volume is especially beneficial for dogs who require multiple CECT examinations to assess disease progression and monitor the response to therapy.^29^ A limitation of CM dose reduction would be increased image noise, owing to the beam‐hardening effect with increasing patient body weight, and to compensate for this, either an increased dose of iodine or an increased X‐ray tube current may be needed.^30^

The similar change in heart rate and blood pressure before and after CM administration in the TBW‐ and LBW‐based protocols was unexpected, particularly given that LBW dosing permitted a reduction in iodine per kg TBW ranging from 8% to 37%. This could indicate that the degree of change in heart rate and blood pressure is not correlated with the dose of administered iodinated contrast or could represent a Type 2 error due to insufficient sample points to demonstrate small differences in these physiologic parameters.

In one dog, there was marginally lower arterial phase attenuation (presented as negative CE) in the portal vein for the TBW dose protocol and spleen for the LBW dose protocol (See Supporting Information S2). This result was not expected and could be due to volume averaging or the inclusion of fat or fluid in the ROI postcontrast. The expected lower CE of the liver and portal vein during the arterial phase would explain the lack of significant difference between their enhancement in the TBW and LBW dosed studies.

A limitation to this study is the small number of dogs derived from a clinical population, with variable time between both CT studies. Small numbers may introduce Type 2 error, particularly in relation to failure to detect a significant difference in physiologic parameters with LBW dose reduction and failure to detect a significant relationship between iodine dose and organ enhancement for most organs with linear regression analysis. Due to the variable time frame between the acquisition of studies by TBW and LBW dosing, organ attenuation may have been affected by various disease processes. Attenuation may also be affected by variations in cardiac function and cardiac output associated with the use of different anesthesia protocols. Cardiac output is a key patient‐related factor affecting CE in humans; when cardiac output is reduced, the distribution of contrast is prolonged, delaying peak enhancement of both vessels and organs.^2^ Another limitation is that the absence of renal disease was only assessed by the lack of recorded relevant clinical signs and normal results of routine serum biochemistry and urinalysis. Additional more invasive and/or expensive tests, such as symmetric dimethylarginine concentrations, renal biopsy, or renal clearance of inulin, were not consistently performed.

This pilot study supports the use of a reduced contrast dose in obese dogs for CECT of the abdomen by dosing according to LBW. Specifically, findings from the current study provided evidence that an intravenous contrast dose reduction for abdominal CT may be beneficial, particularly for overweight dogs, while maintaining images of diagnostic quality. Visual evaluation of studies showed that LBW dosing still produced fair to excellent CE of organs, with studies expected to remain of diagnostic quality despite a reduction in CM dose. Reduced variability in organ enhancement seen with LBW dosing of CM may also prove beneficial for future studies relying on quantification of CE differences to classify disease types and presents an avenue for further investigation. The relationship between organ enhancement and CM dose seems to not be simply linear, however, with hidden compensating effects proposed in the human literature.^25^ Future larger studies are needed to determine the ideal CM dose per LBW to achieve adequate liver enhancement in dogs while reducing the overall iodine load and achieving satisfactory image quality.

## LIST OF AUTHOR CONTRIBUTIONS

Category 1

- Conception and Design: Milne, Mansfield, Tyrrell, Kan
- Acquisition of the Data: Kan
- Analysis and Interpretation of Data: Kan, Milne

Category 2

- Drafting the Article: Kan, Milne
- Revising Article for Intellectual Content: Kan, Milne, Mansfield, Tyrrell

Category 3

- Final Approval of the Completed Article: Milne, Mansfield, Tyrrell, Kan

Category 4

- Agreement to be accountable for all aspects of the work ensuring that questions related to the accuracy or integrity of any part of the work are appropriately investigated and resolved: Kan, Milne, Tyrrell, Mansfield

## CONFLICT OF INTEREST

The authors have declared no conflict of interest.

## EQUATOR NETWORK DISCLOSURE

The authors followed STROBE‐VET reporting guidelines. None of the study findings were previously presented at a scientific meeting and/or published in an abstract.

## Supporting information

Supplementary 1: Descriptive data of dogs (n = 12) when dosed according to TBW and LBW. Significance based on a paired sample t test.

Supplementary 2: Contrast enhancement values for major organs and vessels of the 12 dogs with contrast doses according to TBW. HU, Hounsfield Units
