# Supplementary material for: Lean body weight‐adjusted intravenous iodinated contrast dose for abdominal CT in dogs reduces interpatient enhancement variability while providing diagnostic quality organ enhancement
Source: Vet Radiol Ultrasound. 2022 Jun 10;63(6):719–28. doi: 10.1111/vru.13122 (PMC9796484; doi:10.1111/vru.13122)
Supplement: Supplementary file 1 — Supplementary 1: Descriptive data of dogs (n = 12) when dosed according to TBW and LBW. Significance based on a paired sample t test. [file VRU-63-719-s002.docx]

Supplementary 1: Descriptive data of dogs (n=12) when dosed according to TBW and LBW. Significance based on a paired sample t-test.

|  | TBW dosed scan | LBW dosed scan | Significance |
| --- | --- | --- | --- |
| **Total body weight (kg)** |  |  |  |
| Mean | 33.2 | 33.4 | P = 0.761 |
| Range (Standard Deviation) | 13.0 – 46.0 (13.6) | 11.8 – 54.1 (12.7) |  |
| **Lean body weight (kg)** |  |  |  |
| Mean | 23.5 | 21.7 | P = 0.490 |
| Range (Standard Deviation) | 8.4 – 43.3 (9.7) | 7.5 – 35.0 (7.8) |  |
| **Abdominal fat percentage (%)** |  |  |  |
| Mean | 45.9 | 50.9 | P = 0.129 |
| Range (Standard Deviation) | 15.6 – 15.5 (66.9) | 22.6 – 72.7 (12.6) |  |
