# Supplementary material for: Lean body weight‐adjusted intravenous iodinated contrast dose for abdominal CT in dogs reduces interpatient enhancement variability while providing diagnostic quality organ enhancement
Source: Vet Radiol Ultrasound. 2022 Jun 10;63(6):719–28. doi: 10.1111/vru.13122 (PMC9796484; doi:10.1111/vru.13122)
Supplement: Supplementary file 2 — Supplementary 2: Contrast enhancement values for major organs and vessels of the 12 dogs with contrast doses according to TBW. HU, Hounsfield Units [file VRU-63-719-s001.docx]

Supplementary 2: Contrast enhancement values for major organs and vessels of the 12 dogs with contrast dosed according to TBW. HU, Hounsfield Units

|  | Mean/Median (HU) | Min-Max (HU) | Standard deviation (HU) | Interquartile range (HU) |
| --- | --- | --- | --- | --- |
| **Arterial phase** |  |  |  |  |
| Aorta TBW | 574.9 | 395.8 – 812.1 | 107.7 |  |
| Aorta LBW | 408.2 | 238.1 – 614.3 | 154 |  |
| Liver TBW | 30.0 | 4.9 – 120.6 |  | 41.5 |
| Liver LBW | 16.5 | 3.5 – 53.0 |  | 39.0 |
| Spleen TBW | 28.1 | 8.8 – 86.3 | 24.1 |  |
| Spleen LBW | 14.9 | -1.6 – 39.8 | 14.7 |  |
| Kidney TBW | 353.9 | 12.1 – 534.6 | 139.5 |  |
| Kidney LBW | 239.8 | 71.1 – 336.8 | 69.1 |  |
| Portal Vein TBW | 85.9 | - 1.28 – 371.04 |  | 62.1 |
| Portal Vein LBW | 48.7 | 8.4 – 304.2 |  | 49.3 |
| **Portal venous phase** |  |  |  |  |
| Aorta TBW | 199.4 | 133.7– 247.0 | 33.8 |  |
| Aorta LBW | 140.9 | 101.6 – 191.4 | 37.4 |  |
| Liver TBW | 104.3 | 64.8 – 131.0 |  | 33.0 |
| Liver LBW | 55.4 | 34.0 – 140.5 |  | 26.0 |
| Spleen TBW | 39.8 | 19.0 – 68.1 | 17.0 |  |
| Spleen LBW | 21.9 | 5.9 – 47.9 | 17.0 |  |
| Kidney TBW | 253.7 | 171.4 – 390.0 | 55.1 |  |
| Kidney LBW | 172.2 | 86.4 – 219.5 | 40.0 |  |
| Portal Vein TBW | 213.0 | 40.5 – 271.9 | 58.9 |  |
| Portal Vein LBW | 163.1 | 138.9 – 199.5 | 36.8 |  |
